# Dispositional and performance-specific music performance anxiety in young amateur musicians

**DOI:** 10.3389/fpsyg.2023.1208311

**Published:** 2023-07-31

**Authors:** Claudia Spahn, Pia Tenbaum, Anna Immerz, Jesper Hohagen, Manfred Nusseck

**Affiliations:** ^1^Freiburg Institute for Musicians’ Medicine, University of Music Freiburg, Medical Faculty of the Albert-Ludwigs-University Freiburg, Freiburg Center for Research and Teaching in Music, Freiburg, Germany; ^2^St. Marien- und St. Annastift-Hospital, Clinic for Paediatrics and Adolescent Medicine, Medical Faculty Mannheim, University Heidelberg, Ludwigshafen am Rhein, Germany

**Keywords:** performance science, music performance anxiety, self-efficacy, coping, performance quality, amateur musicians

## Abstract

**Introduction:**

Research on Music Performance Anxiety (MPA) among amateur musicians is of great interest due to inconsistent results in literature. In addition, amateur music represents an important part of musical culture in Germany. Accordingly, the performance experiences of young wind players represent a relevant issue for research and musical practice.

**Methods:**

In the present study, 67 young amateur musicians of a brass choir were examined. Using two different questionnaires, both the dispositional MPA (K-MPAI) and the performance-specific MPA during a joint concert (Performance-specific Questionnaire for Musicians, PQM) were assessed. The PQM measures the symptoms of MPA, functional coping with MPA and self-efficacy before, during and after a specific performance. The PQM was completed by the musicians *via* an app directly after the concert.

**Results:**

Results showed that about 90% of the young amateur musicians had a low dispositional MPA, but about 10% showed high values. For the concrete performance, however, musicians with high dispositional MPA also experienced a very moderate to low MPA in the concert. On average, the musicians were quite nervous before the performance. After the performance, they showed low levels of MPA. Three types of MPA found in previous studies could be confirmed among the amateur musicians, with three quarters being assigned to the positive type, showing low levels of symptoms associated with consistently high levels of self-efficacy and positive functional coping.

**Discussion:**

The results provide a differentiated picture of different expressions of MPA in young amateur musicians. They also raise further questions about the correlation between dispositional and performance-specific assessment of MPA in musicians in general.

## 1. Introduction

Music Performance Anxiety (MPA) is a phenomenon that manifests itself in musicians exposing themselves in front of an audience (Spahn, 2012, 2015). MPA is generally described as a state of excitement, which can bring a variety of negative symptoms of stress reaction (Fernholz et al., 2019; Guyon et al., 2020), but can in its optimal level also enhance a performance. Factors concerning the degree of occurring MPA were identified as the size or composition of the audience, the level of demand, and the assessment of the relevance of the performance (Le Blanc et al., 1997; Spahn, 2012; Zimmermann and Louven, 2017; Osborne and Kirsner, 2022). High levels of MPA can lead to chronically debilitating impacts on future performance experiences (Kenny and Osborne, 2006).

Self-efficacy can play a key role in dealing with MPA. A study by McCormick and McPherson suggests that a high level of self-efficacy is a strong predictor of a positive performance experience (McCormick and McPherson, 2003). In a study with 270 Spanish musicians, González et al. (2018) showed that self-efficacy correlated positively with self-reported experiences of performances.

### 1.1. MPA in amateur and professional musicians

Musicians might react differently based on their professional level, with professionals experiencing higher levels of MPA (Osborne and Kenny, 2005). In a sample of 100 musicians (half professionals and half amateurs), the professional musicians showed higher MPA compared to amateur musicians (Castiglionea et al., 2018). In contrast, among Brazilian musicians, professional and amateur musicians seemed to have similar levels of MPA, but the professional musicians showed higher levels of general social anxiety (Barbar et al., 2014). Papageorgi et al. (2013) reported higher levels of MPA in undergraduate musicians than in professionals, interpretating that more performing experience among the professionals might affect the degree of MPA. A study by Sickert et al. (2022) also showed lower mean MPA scores (K-MPAI-R) among professionals compared to amateurs and music students, with the latter having the highest mean scores.

Overall, different study results are found when comparing MPA in professional and amateur musicians. The survey of MPA in different samples, in particular among amateur musicians, therefore seems worthwhile for further research.

### 1.2. MPA in young musicians

MPA in the context of musical performance can be observed throughout childhood and adolescence (Kenny and Osborne, 2006; Patston and Osborne, 2016; Dempsey and Comeau, 2019; Dobos et al., 2019; Barros et al., 2022). It affects up to one-third of musicians during adolescence (Fehm and Schmidt, 2006) and peaks at age fifteen, with less MPA reported with increasing frequency of performance (Osborne and Kenny, 2005). In a sample of 239 students at German music schools between the ages of 7 and 20, it was also observed that MPA increased significantly at ages 13–15 (Spahn, 2011; Nusseck et al., 2015). Papageorgi (2022) found that MPA in adolescents increased between the ages of 15 and 18, and decreased at the age of 19.

Furthermore, a study examining MPA in young musicians found that those who wanted to make music their profession had less MPA than those who could not imagine it or did not yet know it (Osborne and Kenny, 2005). With increasing age, young musicians begin to perceive themselves and their environment in a more differentiated way, so that they learn to deal with their own insecurity during a performance, however, their attitude of entitlement toward musical performances also increases in the course of adolescence (Kenny and Osborne, 2006). Correlations of higher degrees of MPA in young musicians were found with lower self-confidence and with lower performance quality (Ryan, 2005). Young musicians (aged 7–17 years) often reported a worsening of the performance quality in public performances compared to practice situations (Sokoli et al., 2022).

Other findings revealed a statistically significant negative correlation between MPA and music performance self-efficacy in adolescent musicians (Bersh, 2022). For 16-18-year-olds, self-efficacy was related to the experience of public performances, whereas social support was found to be less correlated with self-efficacy (Orejudo et al., 2021). A strong negative relationship between self-efficacy and MPA was also found by Dempsey and Comeau (2019) in young musicians.

In a sample of 410 young classical musicians in Cyprus and the United Kingdom attending junior conservatoires and/or youth orchestras with ages ranged between 12 and 19 years and a mean of 15.33 years, Papageorgi (2021) performed a cluster analysis on the values of MPA. The analysis revealed three types of MPA with (Cluster 1) moderate anxiety, less intrinsic motivation for learning and low self-efficacy, with (Cluster 2) high anxiety, high MPA and low self-efficacy, and with (Cluster 3) less anxiety, high motivations and high self-confidence. 11% of the musicians experienced high levels of MPA, where 20% were in the low MPA and 69% in the average MPA cluster. Approximately 60% of the cluster variance was explained by individual characteristics such as the susceptibility to anxiety, task-efficacy, and the performance environment.

### 1.3. Dispositional and performance-specific MPA

The understanding of MPA in the field of research primarily refers to the form of MPA as measured by Kenny’s questionnaire (K-MPAI, Kenny, 2009, 2011). The experience of MPA here means a person’s average experience of MPA over an extended period of time. In distinction to this form – for which we propose the term dispositional MPA – MPA can be surveyed with respect to a specific performance – the so-called performance-specific MPA. Other authors also recommend to differentiate between the dispositional level of MPA as a trait component and the occurrence in a concrete performance situation (Papageorgi et al., 2007) and to investigate how the relationship between MPA and self-efficacy is affected in concrete performances (Bersh, 2022).

Trait anxiety was strongly associated with a higher degree of dispositional MPA (Kenny et al., 2004; Papageorgi et al., 2007). Musicians with high levels of dispositional MPA also showed to have higher state anxiety, psychological distress and negative self-assessments compared to musicians with low dispositional MPA levels (Studer et al., 2012; Guyon et al., 2020). However, in a questionnaire survey of 320 professional and student musicians, the dispositional MPA was not a clear indicator for experienced distress, but can also influence perceived performance boosts and confidence (Simoens et al., 2015).

Investigating a concrete performance situation, the Performance-specific Questionnaire for Musicians (PQM, Spahn et al., 2016) considering the experience of MPA at the time directly before a performance, during a performance and after a performance has been developed. The questionnaire needs to be filled in immediately after a performance and addresses three aspects of MPA, i.e., occurring symptoms of MPA, the functional coping with MPA and the self-efficacy for each time point. In a sample of 532 musicians including professional orchestra musicians, amateur orchestra musicians and amateur choir singers, three different types of performance-specific MPA were found (Spahn et al., 2021). Musicians of Type 1 had few symptoms of MPA, high functional coping with MPA and high self-efficacy throughout the performance, indicating healthy and good experiences of the performance. Type 2 describes musicians who began the performance with relatively high symptoms of MPA that reduced after the performance. They also showed rather high values in functional coping and self-efficacy. The musicians in Type 3 did experience the performance in a more unpleasant way. They began their performance with more symptoms of MPA than in the other types. After the performance, those symptoms of MPA even slightly increased. The values of functional coping and self-efficacy were also lower than in the other types. Nearly half of the musicians were classified in Type 1 and about a quarter each to Type 2 and 3. It was found, that amateur musicians in the sample were more often distributed in Type 1 and 2 whereas professional musicians in Type 3. In addition, self-efficacy seemed to have an important influence on the other two scales and therewith on the experience of the performance.

Papageorgi (2021) formed clusters regarding anxiety, motivation, and self-esteem in the sample of 410 adolescent musicians described in Papageorgi (2022). She also found three clusters, each covering one-third of the sample:

*Cluster 1* – moderately anxious students who show lower levels of motivation and feel ineffective, but maintain their self-esteem; experience of physiological symptoms of anxiety, suggesting that they experienced moderate arousal levels.

*Cluster 2* – highly anxious students who have a negative self-image and are prone to maladaptive MPA;

*Cluster 3* – low anxious students who have a high level of motivation and self-confidence and are prone to adaptive MPA.

Regarding this description, similarities can be found between the clusters in Papageorgi (2021) and the types in Spahn et al. (2021). Thus, musicians in cluster 1 according to Papageorgi show similarities to musicians in type 2 according to Spahn et al., those in cluster 2 to those in type 3, and musicians in cluster 3 to those in type 1.

### 1.4. The current study

In the present study, MPA was investigated in young amateur musicians of two brass choirs. The relevance of the study can be seen in the following points. In view of the studies presented above, the investigation of MPA in young musicians and especially in young amateur musicians seems interesting. Furthermore, in the present study, our research group wanted to extend the previously collected results on performance-specific MPA, collect them on a homogeneous sample of amateur musicians, and make direct comparisons with dispositional MPA.

In addition, young amateur brass players represent a significant group in the musical culture in Germany. 19% of the German population make music in their free time, i.e., there are 14 million amateur musicians. Among the 16–29 years olds, the share is 32%. In southern Germany in particular, there is a traditional and well-established structure of music clubs in which wind instruments are very strongly represented (MIZ 2021^1^). The Bund Deutscher Blasmusikverbände e.V., for example, has 1,000 member clubs and 200,000 amateur musicians^2^. Young wind players in amateur music therefore make up an important part.

To our knowledge, there are only a few studies about the MPA state of brass musicians. These studies investigated MPA in brass players in professional orchestras and found an increased MPA compared to other instrumentalists (Fishbein and Middlestadt, 1988; Cohen and Bodner, 2021). The authors justify their results in relation to the soloistic and playing technique demands in the orchestra. However, these demands are hardly comparable to those of amateur musicians in a brass ensemble.

Moreover, Kenny (2011) did not find any instruction-specific correlation with the development of MPA in her study. Regarding the actual research situation concerning the spectrum and coping with MPA among young musicians, we can summarize that there are many studies examining influences of different social and individual factors on MPA of music students (see also Barros et al., 2022). However, the results about the relationship of age, experience and MPA are not consistent, especially associations between different expertise factors and MPA require further clarification. One reason for that could be the lack of studies investigating MPA of music activities in the leisure sector.

Therefore, this study aims at exploratory investigating the dispositional and performance-specific music performance anxiety of amateur musicians in the context of a joint performance of a brass choir. More specific, we study the performance-specific MPA by comparing and relating current scores with other participant groups, research settings and measures of dispositional MPA. We assume, that these exploratory findings contribute to the discourse of MPA among amateur musicians and introduce some new methodological aspects of interest for the field.

## 2. Methods

### 2.1. Study design

The study involved two groups of musicians in two youth brass choirs. The musicians of each group participated in two big-band weekend workshops each with a joint concert at the end of the weekend. All the musicians were provided with information about the study in advance and at the beginning of the workshop. In order to participate in the study, it was required to sign a consent form. For underage participants, informed consent was obtained from their legal guardians. The ethics committee of the University of Freiburg gave a positive vote for the conduct of this study.

Two surveys took place at two points in time. At the beginning of the first weekend workshop, the participants were asked to answer sociodemographic (age, gender) and music-related (instrument, years of instrumental training) questions and to fill in a first standardized questionnaire about their individual disposition in experiencing MPA. After the joint concert at the end of the second weekend workshop, participants were asked to complete a second standardized questionnaire regarding the experience of MPA related to the just finished performance. The first questionnaire was provided in paper form. The second questionnaire was filled in directly after the concerts and was presented as a smartphone app that the participants either installed on their own device or used on someone else’s device.

### 2.2. Participants

A total of 67 musicians participated in the study. In the first group were 32 musicians, in the second group 35 musicians. The instruments amounted to 43% trombone, 43% trumpet, 9% horn and 5% tuba. Regarding gender distribution, age and years of instrumental training, there were no significant differences between the two groups. Therefore, the participants of both groups were combined into one sample. In this sample (*n* = 67) the percentage of female musicians was 58%. On average, participants trained their instrument for 9.5 years (range: 5–17 years, SD = 2.86 years).

The mean age of the musicians was 18.5 years (SD = 3.03 years). The range was 13–26 years. The age amounted to 17% 13–15 years, 36% 16–18 years, 36% 19–22 years and 11% over 22 years. This shows a rather normal distribution across the age groups.

After the joint concerts, 58 musicians filled in the second questionnaire. As well as in the sample of 67 musicians, there were still no significant differences between the two groups.

### 2.3. Questionnaires

#### 2.3.1. Kenny-music performance anxiety inventory (K-MPAI)

Because of the age range of the sample between 13 and 26 years, the adult version of the K-MPAI was chosen instead of the version for use with child and adolescent musicians (Osborne and Kenny, 2005). The Kenny-Music Performance Anxiety Inventory (K-MPAI, Kenny, 2009, 2011) is a standardized questionnaire to determine the dispositional degree of MPA. The revised version has 40 items answered on a 7-point Likert scale ranging from 0 = “do not agree” to 6 = “fully agree.” The total scale was used that showed high internal reliability (Cronbach’s alpha = 0.94; Kenny, 2009). A higher score represents higher levels of MPA as well as psychological distress (Kenny, 2011). The questionnaire has been validated and used in numerous research studies and is available in several languages (Kenny, 2023).

The K-MPAI scale ranges between 0 and 240. In a sample of 373 professional orchestra musicians, a mean value of 83.7 (SD = 40.7) was found (Kenny et al., 2012). Using this sample, a comparative analysis with other established clinical screening tests of anxiety and depression was performed to indicate possible cut-off values for high degrees of MPA (Kenny, 2015). The findings suggested a high level of MPA above 104. In the present study, a translated German version of the questionnaire was used and showed a high internal reliability (Cronbach’s alpha = 0.89; *n* = 67).

#### 2.3.2. Performance-specific questionnaire for musicians (PQM)

To measure self-reported MPA considering a particular performance, the Performance-specific Questionnaire for Musicians (PQM, Spahn et al., 2016) was used. The questionnaire requires to be filled in immediately following a performance. It contains a total of 42 items with the first 32 questions addressing retrospectively the times before, during, and after the performance. For each time of the performance, three aspects of MPA were assessed: (1) the functional coping with MPA, i.e., positive activities in handling with MPA (Cronbach’s alpha: before 0.73, during 0.80, after 0.66, *n* = 58), (2) symptoms of MPA (Cronbach’s alpha: before 0.81, during 0.83, after 0.67, *n* = 58), and (3) self-efficacy, i.e., one’s own confidence in performing (Cronbach’s alpha: before 0.71, during 0.77, after 0.83, *n* = 58). For the three scales, similar items were used across the different performance times with the prefaces “A few minutes before the performance…,” “During the performance…” and “Now, after the performance….” Examples of items are for the functional coping “… I could concentrate on my musical performance,” for the symptoms of MPA “… I thought about all the things that could go wrong” and for the self-efficacy “… I could imagine the audience enjoying my performance.” The questions were answered on a 5-point Likert scale (1 = “does not apply” to 5 = “applies very much”). High values in the scales functional coping and self-efficacy indicate better coping and higher self-efficacy whereas high scores in the scale symptoms of MPA give notice of debilitating MPA.

An additional scale with seven items evaluates the self-perceived musical quality of the performance (Cronbach’s alpha = 0.77, *n* = 58). The music-related aspects were rated on a 6-point scale ranging from 1 = “very poor” to 6 = “excellent.”

Specific personal aspects of the performance were queried with three further items. The participants were asked to state the personal importance of the performance on a 4-point scale (1 = “not important” to 4 = “very important”). In another question, the musicians were asked to rate the difficulty of the performance compared to other performances on a 4-point scale (1 = “easy” to 4 = “difficult”). Finally, the general personal difficulty of the concert was assessed on a 5-point scale (1 = “too low” to 5 = “too high”).

### 2.4. Statistics

The analyses were carried out with SPSS 28 (Armonk, NY: IBM Corp). Descriptive statistics include the mean value and the standard deviation (SD) of the mean. A hierarchical cluster analysis (Method: single-linkage between groups; Squared Euclidean Distance) was performed on the K-MPAI scale. With the cluster solution, a k-mean cluster analysis was performed.

To classify the different types of MPA according to Spahn et al. (2021), a k-mean cluster analyses with three clusters were performed with the PQM scales regarding the time before the performance. The percentage of explained variance was calculated with a discriminate analysis. Multivariate analysis of variance (MANOVA) was used for the comparative analysis of the questionnaire scales. On significance, post-hoc analyzes were performed using the Tukey-HSD correction.

Independent t-tests were used for individual comparisons. Nonparametric comparisons were examined using a cross table reporting Pearson’s *χ*^2^. Significant Pearson’s r correlation coefficients were categorized as followed: *r* < 0.3: weak to no correlation, *r* > 0.3 and < 0.5: moderate correlation, *r* > 0 0.5: strong correlation (Cohen, 1988). The level of significance was set to *p* = 0.05.

## 3. Results

### 3.1. Dispositional MPA

#### 3.1.1. Descriptive results of the K-MPAI

In the K-MPAI, measuring the dispositional MPA, the mean value of the whole sample was 85.3 (SD = 27.5). The value did not differ significantly from the mean value of the orchestra musicians (t(57) < 1.0; Kenny et al., 2012) and it was significantly below the cut-off for high degrees of MPA at 104, found by Kenny (2015) in professional orchestra musicians [*t*(57) = −5.18; *p* < 0.001].

Female musicians scored slightly higher (89.5; SD = 25.4) than male musicians (78.9, SD 29.7), but without statistical significance [*F*(1,56) = 2.1; n.s.]. The K-MPAI scale did not correlate significantly with age (*r* = 0.07) nor with the years of instrumental training (*r* = 0.08).

#### 3.1.2. Cluster analysis on the K-MPAI

The cluster analysis on the K-MPAI yielded a three groups solution with a 92% explained variance. One group of 26 musicians (45%) showed quite low K-MPAI values (60.9; SD = 10.1), a second group of 18 musicians (31%) showed medium K-MPAI values (80.4; SD = 7.9), and a third group of 14 musicians (24%) had high K-MPAI values (124.1; SD = 13.3). These groups did not differ significantly in gender, age and years of instrumental training. While the mean values of the first two groups were significantly below the cut-off of 104 (*p* < 0.001), the value of the third group was significantly above this cut-off (*p* < 0.001).

### 3.2. Performance-specific MPA

#### 3.2.1. Descriptive results of the PQM

The results of the PQM refer to the joint performances of the brass choirs. The scales of the PQM assess symptoms of MPA, functional coping and self-efficacy before, during and after the performance (Table 1).

**Table 1:** Mean values with standard deviations (SD) in the PQM scales (range 1–5) of the young amateur musicians (*n* = 58) with statistical analysis (repeated measure ANOVA).

	Before the performance	During the performance	After the performance	Statistics
Symptoms of MPA	2,3 (0,92)	2,3 (0,94)	1,6 (0,58)	*F*(1,57) = 14.8; *p* < 0.001
Functional coping	4,2 (0,76)	4,0 (0,83)	4,4 (0,58)	*F*(1,57) = 4.75; *p* = 0.033
Self-efficacy	4,0 (0,73)	4,1 (0,68)	4,0 (0,84)	*F*(1,57) < 1.0; n.s.

Overall, the results of the PQM scales show that the musicians had a high functional coping with MPA and a high self-efficacy. In the scale symptoms of MPA, the musicians tended to have rather high values before and during the performance, which decreased significantly to a low level after the performance [*F*(1,57) = 14.8; *p* < 0.001; Post-Hoc: before/during to after: *p* < 0.001]. In the course of the performance, functional coping was high before, decreased during the performance and increased significantly again after the performance [*F*(1,57) = 4.75; *p* = 0.033; Post-Hoc: during to after: *p* = 0.010]. The self-efficacy scale did not change significantly across the performance.

#### 3.2.2. Classification of types of MPA

**Figure 1:**
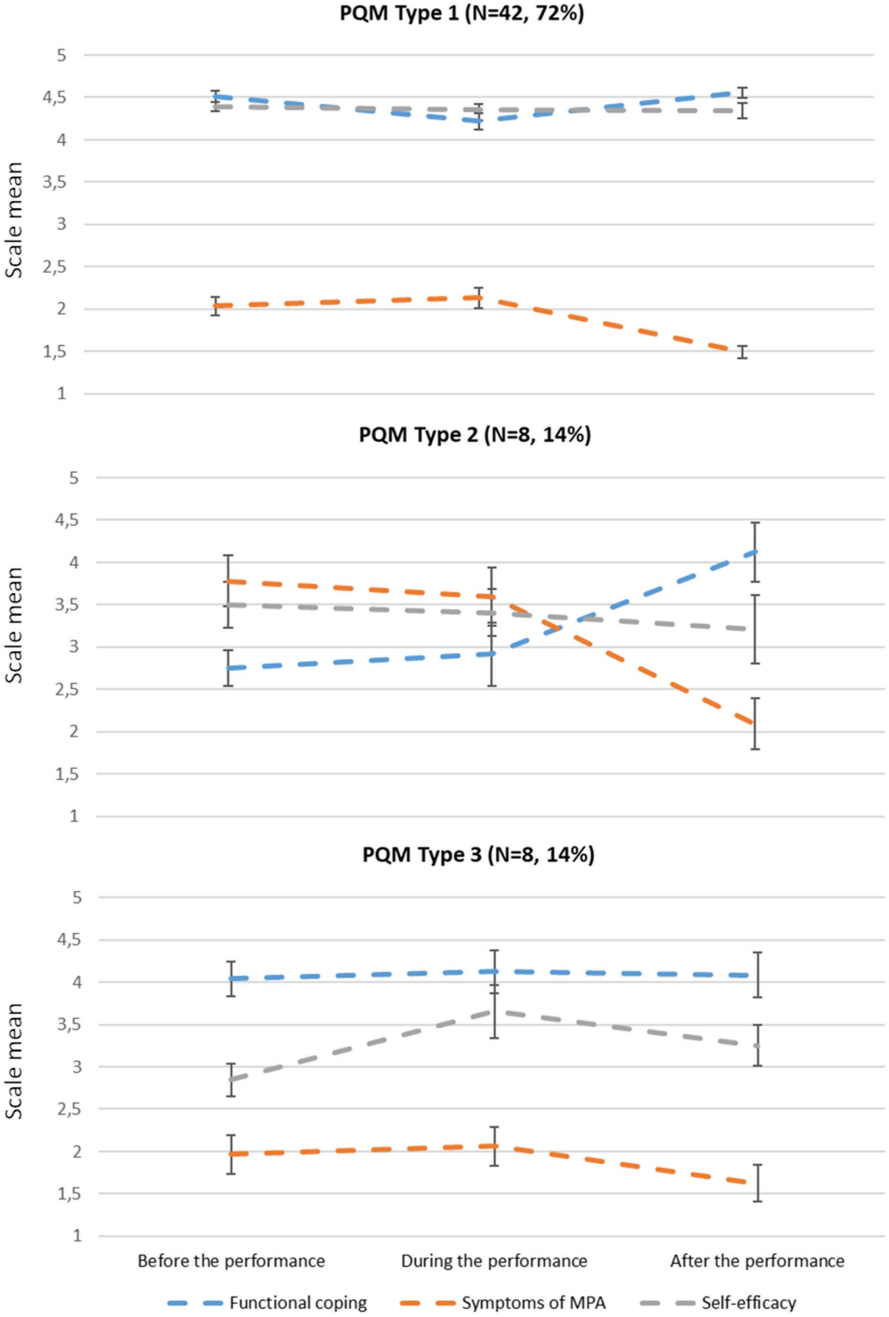
Mean values (error bars represent the standard error of the mean) in the PQM scales by PQM Type 1, 2 and 3 according to Spahn et al. (2021), among young amateur musicians (*n* = 58).

Regarding the course of the PQM scales over the performance, the k-mean classification of participants with three clusters according to Spahn et al. (2021) resulted in a distribution of 42 musicians (72%) in Type 1, eight musicians (14%) in Type 2, and eight musicians (14%) in Type 3 (Figure 1).

Compared with the mean scores of the PQM scales of the three Types in Spahn et al. (2021), the symptoms of MPA scales before and during performance were higher in *Type 1* in this study. After the performance, the values have adjusted to a similar low level as in Spahn et al. (2021).

*In Type 2*, the course of the PQM scales was rather comparable to Spahn et al. (2021), with significant increasing functional coping over the performance [Friedman-Test; *χ*^2^(8,2) = 7.52; *p* = 0.023] and rather high symptoms of MPA before the performance that significantly decrease after the performance [Friedman-Test; *χ*^2^(8,2) = 13.0; *p* = 0.002]. However, the values in the functional coping before and during the performance were much lower and the symptoms of MPA were much higher in this study.

The low score in the self-efficacy in *Type 3* was similar to Spahn et al. (2021). In contrast to the increase of the symptoms of MPA after the performance of Type 3 in Spahn et al. (2021), the mean value remained the same here [Friedman-Test; *χ*^2^(8,2) = 1.36; n.s.] and was at a similar level of the other two Types.

#### 3.2.3. Comparison of MPA type classifications

The shown classification of the MPA types were performed with the distribution parameters of the discriminant analysis in Spahn et al. (2021). When performing a separated k-mean clustering on this sample, very similar properties of each cluster according to the types in Spahn et al. (2021) were found. The comparison between the two MPA type classifications showed 82% agreement. Therefore, the type distribution according to Spahn et al. (2021) was also reliable to this sample.

#### 3.2.4. Self-rated quality, importance and difficulty of the performance

The quality of the performance was rated by the musicians with an average of 4.3 (SD = 0.5), which means – on a scale range of 1 to 6 – a rather good assessment. The importance of the performance was valued with 3.3 (SD = 0.7), describing the performance – on scale range of 1 to 4 – as a quite important one. The difficulty of the performance compared to other performances was judged to be just right (2.9; SD = 0.5; scale range of 1 to 5) and the general performance difficulty was experienced as easy but not too easy (2.1; SD = 0.7; scale range of 1 to 5).

There were significant correlations between the rated performance quality and the symptoms of MPA, with lower symptoms associated with higher ratings (before: *r* = −0.38, during: *r* = −0.19, after: *r* = −0.33) and the self-efficacy (before: *r* = 0.39, during: *r* = 0.37, after: *r* = 0.34). Another significant correlation was found between the rated performance quality and the importance of the performance (*r* = 0.34).

### 3.3. Relationship between dispositional and performance-specific MPA

A comparison between the three K-MPAI cluster groups and the PQM Types 1–3 revealed no significant difference in distribution [χ^2^(57) = 6.48; n.s.].

#### 3.3.1. Correlations between K-MPAI and PQM values

Correlation analyses between the K-MPAI value of the total score and the PQM scales showed significant correlations with the symptoms of MPA scales before and after the performances, the functional coping scale after the performance, and the self-efficacy scales at all times of the performance (Table 2). No significant correlations were found for the importance of the performance, for the compared and general difficulty, and for the self-rated quality of the performance.

**Table 2:** Correlations between the K-MPAI and the PQM scales (*p < 0.05; **p < 0.01) and mean values with standard deviation (SD) for the different PQM scales by K-MPAI cluster Groups 1, 2 and 3 with significant differences between the groups (bold, significant effects; n.s., not significant).

PQM	Correlations with the K-MPAI (total score)	Group 1 (*n* = 26) Low K-MPAI mean (SD)	Group 2 (*n* = 18) Medium K-MPAI mean (SD)	Group 3 (*n* = 14) High K-MPAI mean (SD)	Statistical difference between the Groups 1, 2 and 3
Importance of the performance	−0.13	3.4 (0.6)	3.2 (0.5)	3.1 (0.9)	n.s.
Compared difficulty	0.22	2.0 (0.7)	2.1 (0.8)	2.3 (0.7)	n.s.
General difficulty	0.13	2.9 (0.5)	3.1 (0.4)	2.9 (0.6)	n.s.
Quality of the performance	−0.16	4.3 (0.4)	4.4 (0.3)	4.2 (0.6)	n.s.
PQM scales					
Symptoms of MPA (before)	0.26*	2.2 (0.7)	2.2 (1.1)	2.5 (0.9)	n.s.
Functional coping (before)	−0.15	4.3 (0.8)	4.1 (0.8)	4.2 (0.7)	n.s.
Self-efficacy (before)	−0.39**	4.3 (0.5)	4.1 (0.6)	3.6 (0.9)	***F*(2,55) = 4.23; *p* = 0.019**
Symptoms of MPA (during)	0.10	2.3 (0.9)	2.3 (0.9)	2.4 (1.0)	n.s.
Functional coping (during)	−0.06	4.0 (0.9)	4.0 (0.9)	4.1 (0.6)	n.s.
Self-efficacy (during)	−0.30*	4.3 (0.6)	4.1 (0.6)	3.9 (0.6)	n.s.
Symptoms of MPA (after)	0.34**	1.5 (0.5)	1.6 (0.5)	1.9 (0.7)	***F*(2,55) = 3.11;** *p* = 0.052
Functional coping (after)	−0.38**	4.7 (0.3)	4.3 (0.5)	4.2 (0.8)	***F*(2,55) = 5.06; *p* = 0.010**
Self-efficacy (after)	−0.42**	4.3 (0.6)	4.0 (0.8)	3.5 (1.0)	***F*(2,55) = 4.77; *p* = 0.012**

To investigate differences in the PQM scales between the three different groups of dispositional MPA, all PQM scales were compared between the three K-MPAI cluster groups (Table 2). The multivariate analysis showed significant differences in the self-efficacy scale before the performance between group 1 (low MPA) and group 3 (high MPA; *p* = 0.015), in the functional coping scale after the performance between group 1 and 3 (*p* = 0.017), and in the self-efficacy scale after the performance between group 1 and 3 (*p* = 0.010).

## 4. Discussion

In the present study, the results showed that about 90% of the young amateur musicians had a low dispositional MPA, but about 10% showed high values. For the concrete performance, however, musicians with high dispositional MPA also experienced a very moderate to low MPA in the concert. On average, the musicians were quite nervous before the performance. After the performance, they showed low levels of MPA. Three types of MPA found in previous studies could be confirmed among the amateur musicians, with three quarters being assigned to the positive type 1, showing low levels of symptoms associated with consistently high levels of self-efficacy and positive functional coping. In the following, we discuss the results on dispositional and performance-specific MPA and a relationship between the two in light of the existing literature.

### 4.1. Dispositional MPA in young amateur musicians

The young amateur musicians studied here showed, on average, a rather low dispositional MPA, which is below the cut-off of the K-MPAI established by Kenny. The values of professional orchestra musicians by Kenny (2015) were used as a comparison. The results are in agreement with those of Castiglionea et al. (2018), but different from Barbar et al. (2014) and Papageorgi et al. (2013), who found comparable or higher values for MPA in amateur musicians compared to professional musicians.

A direct comparison of the present results is provided by the study of Sickert et al. (2022), in which the K-MPAI was also used in amateur musicians. In this German sample of 122 amateur musicians (age range: 19–71 years, mean 35.3 years, SD = 15.8 years), a mean value of 98.5 (SD = 40.9) was found (Sickert et al., 2022). The mean value in the present study is significantly lower at 85.3 (SD = 27.5; age range: 5–17 years, mean 9.5 years, SD = 2.86 years). Comparing the two studies, the different age structure of the samples is striking: in our study, there is a lower age range and a younger average age than in the study by Sickert et al. (2022). The extent to which these sample differences explain the differential MPA is difficult to interpret and should be the subject of further investigation.

The often found higher MPA in girls compared to boys (Osborne and Kirsner, 2022), was also seen in our sample, but did not become statistically significant. Similarly, no correlations were found between K-MPAI with age or years of instrumental training in consent with Dobos et al. (2019), but in opposite with other studies (Osborne and Kirsner, 2022). The results are difficult to include in this particular discussion. However, the findings may be caused due to the fact of the young age of the sample and the low variances in age and instrumental training.

To classify the large variance of dispositional MPA within the sample, a cluster analysis was performed on the K-MPAI scale. It yielded a clear solution of three different groups of the degree of MPA. The analysis found that about half of the young amateur musicians rated their MPA as quite low, about one third as medium and about a quarter of the musicians as high. The values for the latter group were significantly above the cut-off of Kenny (2015) in the K-MPAI.

The groups in our sample with low, medium, and high MPA did not differ significantly in gender, age, and years of instrumental training. Papageorgi (2022) was able to elucidate 60% of the variance in MPA by variables that related to individual characteristics such as high anxiety, task-efficacy, and the performance environment. In this context, the results of our study on MPA with regard to joint performance are particularly interesting, especially since here external influencing factors such as the musical task and the performance environment were the same for the musicians and thus person-related, individual factors must be the decisive influencing factors on the experience of MPA.

### 4.2. Performance-specific MPA in young amateur musicians

In our study, we had the opportunity to survey the course of MPA before, during, and after the final concert. The focus was on how strongly the musicians experienced the symptoms of MPA, how well they were able to cope with MPA and how high their self-efficacy was.

Regarding the joint concert, the musicians showed to have on average high functional coping and self-efficacy over the whole performance. However, they had rather high values in the symptoms of MPA scales before and during the performance. The symptoms of MPA decreased significantly to a low level after the performance.

Because the MPA symptoms did not correlate with age or years of instrumental training, they may be related to personal characteristics such as dispositional MPA. Interestingly, the K-MPAI total score showed a significant correlation with the symptoms of MPA after the performance, but only a weak, not significant correlation with the symptoms of MPA before and no relevant correlation with the symptoms of MPA during the performance. This leads to the assumption that the dispositional MPA seems not to be related with the performance specific MPA. This can be underlined by the findings the correlations with the performance quality scale. Since the K-MPAI is not correlated with the rated performance quality, the PQM scales were. This situational distress has more influence on the performance quality as the dispositional MPA.

#### 4.2.1. Classification of types of MPA

Spahn et al. (2021) described three types of courses in terms of the interplay of symptoms of MPA, functional coping with MPA and self-efficacy related to a specific performance. In the present sample of amateur musicians, a similar cluster analysis was performed in order to compare the results with the typology by Spahn et al. (2021). The types 1, 2 and 3 are based on the different constellations of symptoms of MPA, functional coping with MPA and self-efficacy before the performance:

Musicians of *type 1* show low symptoms of MPA before the performance and high functional coping and self-efficacy at the same time. This initial constellation was shown by almost three-fourths of the amateur musicians in our sample, whereas only about half of the sample with professional and amateur musicians by Spahn et al. (2021) were of type 1. In the further course during and after the performance, the favorable constellation of symptoms of MPA, functional coping and self-efficacy is maintained in type 1, and symptoms of MPA are low after the performance. This was particularly the case for musicians in our sample. Thus, a favorable course persists in three-quarters of the amateur musicians belonging to type 1.

*Type 2* musicians, in contrast to type 1, show high symptoms of MPA before performance, but also high self-efficacy at the same time. In our sample, functional coping was only moderate in type 2 musicians before the performance, but increased significantly until after the performance. According to the course of type 2, during and after the performance the symptoms of MPA decrease, which was significantly the case in our sample. Type 2 accounted for 14% of the amateur musicians in our sample.

Musicians of *type 3* show a rather low self-efficacy with moderate symptoms of MPA. In our sample, functional coping was strong among type 3 musicians. In the course, self-efficacy increased slightly until after the performance. In the musicians of type 3, a low value of symptoms of MPA was found in our sample with an overall positive constellation with self-efficacy and functional coping. Type 3 accounted for another 14% of the amateur musicians in our sample.

With regard to the different constellations before the performance, the three types found in the present sample of amateur musicians confirm the prescribed types by Spahn et al. (2021). What is striking in the present sample is the positive constellation present in all three types after the performance.

In comparison to the cluster analysis on young classical musicians of Papageorgi (2022), the classification in three MPA clusters was very similar. However, our sample contained twice as many young musicians with high dispositional MPA, but also more than twice as many with low MPA. Although the percentage distribution of low and high dispositional MPA differs between Papageorgis and our sample, we find that there is an interesting commonality of finding distinct subgroups in the expression of dispositional MPA among the adolescent and young amateur musicians. In our sample, which is rather homogeneous in terms of age, playing practice, and instrument, this result appears to us as particularly remarkable. With regard to dispositional MPA, different individual prerequisites for performance are thus present within the musicians.

Even though the typologies in both studies Spahn et al. (2021) and Papageorgi (2021) are not identical, they indicate that patterns can be found and described with regard to the factors symptoms of MPA, coping with MPA, general anxiety, self-esteem and self-efficacy. In this respect, the typology of the present sample of amateur musicians confirms the described typologies on the one hand and makes clear on the other hand that they exist independently of age and of professional or amateur status and show only gradual differences.

### 4.3. Dispositional and performance-specific MPA

For the assessment of MPA in our amateur musicians, we asked ourselves what relationship could be described between dispositional MPA and experienced MPA in the musicians’ joint concert. An important result was that no statistical correlation was found between the groups low, medium, and high in dispositional MPA and the types 1, 2, and 3 in the PQM-Scales over the performance.

Significant correlations between dispositional MPA and performance-specific scales of the PQM were found consistently for self-efficacy, both before and during and after performance. In our view, this validates both questionnaire instruments, especially since items on self-efficacy are included in the K-MPAI. In addition, it was striking that correlations between dispositional MPA in the K-MPAI with the PQM scales correlate after performance and not - with the exception of self-efficacy – with the PQM scales before and during performance.

The results are not easy to classify. One interpretation could be that in the situation after the performance personality-related factors, as depicted by the K-MPAI, gain stronger influence, whereas before and during the performance the adrenergic reaction associated with the performance is more prominent. However, the relationship between the dispositional MPA, which surveys enduring experiences with performance situations, and the MPA related to a specific performance raises fundamental questions that, in our view, are not specific to the group of amateur musicians.

Overall, the possible assumption that musicians with high levels of dispositional MPA might also show high levels of MPA in certain performances could only be partially confirmed. The lack of a significant distribution pattern between the K-MPAI groups and the PQM Types showed that there is no overall relation between both. Musicians with high dispositional MPA can also have experienced a very moderate to low MPA in the concert. Our data show that the dispositional MPA seems to be less associated with the degree of functional coping or the symptoms of MPA in a particular performance, especially before and during the performance.

Furthermore, the distinction between dispositional and performance-specific MPA seems to be a current topic in recent publications (Spahn et al., 2021 Papageorgi, 2022). The distinction of these forms of MPA could be an important topic for future research.

The correlation between K-MPAI and the self-judged quality of the performance seems to be rather difficult. The results showed that between MPA and the performance quality there was no correlation. Ryan (2005) suggested a decreasing performance quality due to higher MPA. The finding indicate that the self-assessed quality of the performance is independent from the general MPA. However, it seems to be more related to the particular situation of the performance. Thus, the quality rating significantly correlated with the symptoms of MPA before the performance (*r* = −0.38) but this scale did not correlate with the K-MPAI.

The application of these results for practice seems to us to be particularly important with regard to a differentiated perception of performance experiences. In an active and resource-oriented view and analysis of a musical performance can lie the chance to develop a dynamic and realistic self-concept as a musician.

## 5. Limitations of the study

The limitations of the study are mainly due to the relatively small sample size. However, this disadvantage is partially offset by the homogeneity of the sample with respect to the instrument and age range. The study setting involved only one joint performance, however, even here there was a great consistency in the external factors, as all musicians performed under the same conditions.

The results and implications can only be generalized with reservations, especially since we are dealing with a specific group of amateur musicians in a brass choir. Overall, we consider the results of the study to be preliminary. The conclusions drawn here provide numerous starting points for further replication studies.

## 6. Conclusion

Young amateur musicians in our study showed individual differences with respect to the expression of dispositional MPA. Pre-performance symptoms of MPA were also high and pre-performance self-efficacy was low in some musicians. The results provide a differentiated picture of different expressions of MPA in young amateur musicians.

The present study provides new insights on MPA in a specific performance among young brass musicians in amateur music. This seems of particular importance given the few studies to date on this group of musicians on the topic of MPA. Overall, the results indicate that MPA plays a relevant role among these musicians and that it is worthwhile to keep the topic of coping with MPA in this group of musicians in mind and to give practical recommendations if needed.

## Data availability statement

The raw data supporting the conclusions of this article will be made available by the authors, without undue reservation.

## Ethics statement

The studies involving human participants were reviewed and approved by Ethics Committee of the University Clinic Freiburg. Written informed consent to participate in this study was provided by the participants’ legal guardian/next of kin.

## Author contributions

PT did mainly the data collection. CS and MN performed the statistical analyses. All authors contributed to the article and approved the submitted version.

## Funding

We acknowledge support by the Open Access Publication Fund of the University of Freiburg.

## Conflict of interest

The authors declare that the research was conducted in the absence of any commercial or financial relationships that could be construed as a potential conflict of interest.

## Publisher’s note

All claims expressed in this article are solely those of the authors and do not necessarily represent those of their affiliated organizations, or those of the publisher, the editors and the reviewers. Any product that may be evaluated in this article, or claim that may be made by its manufacturer, is not guaranteed or endorsed by the publisher.

## Footnotes

^1^ https://miz.org/de/dokumente/musikindustrie-in-zahlen-2021

^2^ https://www.bdb-akademie.com/verband/mitglieder/
